# Hybrid 3D printed-paper microfluidics

**DOI:** 10.1038/s41598-020-75489-5

**Published:** 2020-10-27

**Authors:** Arthur Zargaryan, Nathalie Farhoudi, George Haworth, Julian F. Ashby, Sam H. Au

**Affiliations:** Department of Bioengineering, Imperial College London, London, SW7 2AZ UK

**Keywords:** Fluidics, Biomedical engineering

## Abstract

3D printed and paper-based microfluidics are promising formats for applications that require portable miniaturized fluid handling such as point-of-care testing. These two formats deployed in isolation, however, have inherent limitations that hamper their capabilities and versatility. Here, we present the convergence of 3D printed and paper formats into hybrid devices that overcome many of these limitations, while capitalizing on their respective strengths. Hybrid channels were fabricated with no specialized equipment except a commercial 3D printer. Finger-operated reservoirs and valves capable of fully-reversible dispensation and actuation were designed for intuitive operation without equipment or training. Components were then integrated into a versatile multicomponent device capable of dynamic fluid pathing. These results are an early demonstration of how 3D printed and paper microfluidics can be hybridized into versatile lab-on-chip devices.

## Introduction

Microfluidic paper analytical devices (μPADs) are well suited to point-of-care testing due to their portability, compatibility with colourimetric analyses, and the ability to passively drive fluids by capillarity^1^. These attractive features are a result of paper’s ability to retain and transport liquids within its porous structure, which also enables the incorporation of hydrophobic substrates during μPAD fabrication^2,3^. However, paper’s porosity also causes some drawbacks. μPADs have difficulty (a) storing and dispensing large volumes without significant fluid losses and (b) starting, stopping and timing fluid flows. Time-delayed control elements have been developed to provide some fluidic control on μPADs including valves that incorporate wax barriers dissolved by solvents^4,5^**,** swellable polymers^6,7^, dissolvable sugar barriers^8^, porous hydrophobic barriers^9^ and glass fibre dissolvable bridges^10^. Because these structures rely heavily on material-liquid interactions, they are incompatible with many solvents^11^, require precise calculations to optimize timings for each application and most importantly, are single-use (i.e. once these valves are actuated, they cannot be returned to their original states). Mechanically actuated valves have also been developed such as layered push-to-contact paper valves^12^ and compressive sponge actuators^6^ but these are irreversible once actuated. Other μPAD valve modalities rely on external magnetic fields^13,14^ or local heating via thin film resistors^15^. However, the complexity and requirement for electronic equipment may hinder the applicability of these formats, especially in low-resource settings or with untrained operators. We currently do not have a user-friendly methods for liquid storage and pathing in paper-based microfluidic devices. Such methods would allow μPADs to be used in increasingly complex and diverse applications.

3D printing is a versatile fabrication technique that deposits material into three-dimensional space. This allows for the formation of microfluidic structures that are difficult to fabricate with standard photolithography such as rounded cross-section or complex curvilinear microchannels^16,17^ and adapters to commonly used connection ports such as luer locks^18^ and O-ring seals^19^. This technology has many other attractive features for microfluidics^20–22^, including its ability to print biocompatible plastics^20^, hydrogels, live cells^23^, metals^24^, sugars^25^, glass^26^, its simple integration with analytical chemistry modalities^27^, and its ability to iteratively update complex prototypes without the need for photomasks. 3D printing also benefits from ongoing technological improvements that decrease costs, printing times and achievable resolutions. 3D printed Microfluidics however require external methods of fluid manipulation and have difficulty incorporating spatial patterning required for many biochemical and visual readouts. Overcoming these drawbacks would greatly expand the applicability of 3D printed devices for use in resource-limited settings.

In this work, we present a practical method to fabricate hybrid microfluidic devices that incorporate both 3D printed and paper-based elements to simultaneously addresses the advantages and limitations of each technology. This method operates by 3D printing directly onto standard laboratory filter paper using widely-available commercial fused deposition modelling (FDM) printers. To demonstrate the capabilities of this technology, we designed finger-actuated reservoirs and reversible mechanical valves that can be intuitively operated by untrained users. Finally, these elements were then integrated into versatile devices that demonstrated fluidic control required for μPAD channel washing and re-use. These prototypes represent an advancement towards readily-accessible yet versatile hybrid microfluidic devices.

## Materials and methods

### 3D-printed paper channels

All components and assemblies were designed using SolidWorks 2019 (DSSC, USA) and exported in STL format into CURA (Ultimaker BV, Netherlands) where they were sliced and exported in GCode format. STL files and their editable SolidWorks files for components described in this work are freely available in the Supplementary information.

Hybrid channels were formed by depositing 2.85 mm Polypropylene (PP) filament (Formfutura, Netherlands) directly onto Whatman Grade 1 125 mm diameter round filter paper (Fischer Scientific, UK) taped directly onto the build plate using an Ultimaker 2 + 3D printer (3D Gbire, UK). The bed-level of the 3D printer was precalibrated to account for the extra thickness of filter papers. For evaluating channel barrier integrity via liquid retention tests, we printed simplified 10 mm diameter circular devices. A feature height of 0.50 mm and widths of 1–5.0 mm were printed with slicing parameters: 0.1 mm layer height, 0.4 wall thickness, 0.6 bottom/top thickness, no build plate adhesion or support and a 60% infill.

Printed channels were then baked at 150–200 °C for 15–45 min (optimal 170 °C for 45 min) (Severin TO 2034, Severin Elektrogeräte GmbH, Germany) controlled by a beta Layout Reflow Controller V3 Pro1. The reflow controller was set with pulse width modulation to achieve temperatures no higher than 170 °C to prevent leeching of filament components. After baking, devices were securely flattened and allowed to cool for 5 min. Before operation, the undersides of devices were coated with Jaxon white wax pastel (Honsell GmbH, Germany).

Hybrid channels were tested by pipetting 500 µL of ~ 1% (v/v) blue food colouring in water solution onto one end of hybrid channels. Solution was flowed over 60 min, and channels, where the dye solution crossed the printed barriers at any location, were classified as having lost barrier integrity. Barrier integrity was calculated as the percentage of the tested devices that leaked fluid at any location across hydrophobic barriers.

**Figure 1:**
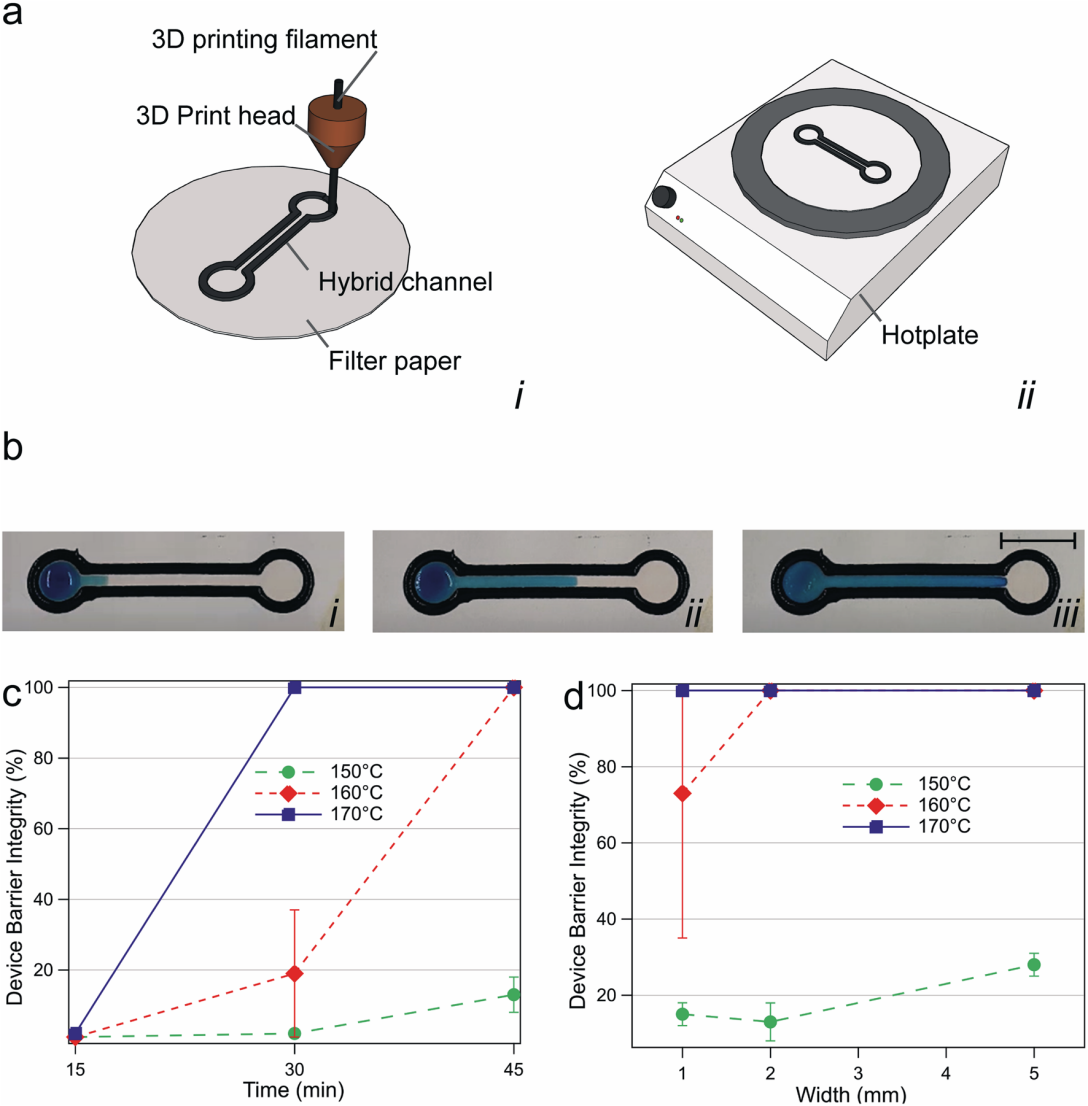
Hybrid device fabrication and integrity. (**a**) PP filaments deposited directly onto filter paper and baked to create hydrophobic barriers (not to scale). (**b**) Flow of dye solution on completed device. (**c**) Barrier integrity of fabricated devices with 2 mm barrier widths for bake durations of 15–45 min (**d**) Barrier integrity of devices for bake durations of 45 min at barrier widths of 1–5 mm. Temperatures tested: 150 °C (green dot), 160 °C (red diamond) and 170 °C (blue square). Error bars represent standard deviation, N = 3. Scale bar: 10 mm.

Based on barrier retention tests, channels described in the remainder of this work were printed with 2 mm channel widths and baked at 170 °C for 45 min unless specified otherwise. To test hybrid channel operation, we then printed “dogbone”-shaped assemblies consisting of 40.0 mm × 3.0 mm straight channels connected on both ends to 10.0 mm diameter circular zones with feature height of 0.50 mm and widths of 1–5.0 mm as described above (Fig. 1a).

### Valves

Valves consisted of 2 components printed separately: “body” and “bridge”. Components were fabricated as described above, but printed using Blue or Yellow 2.85 mm diameter Polylactic Acid (PLA) filament (Innofil 3D B.V., Netherlands). Slicing parameters: 0.2 mm layer height, 0.76 wall thicknesses, 0.8 bottom/top thickness, 40% infill and no build plate adhesion or support. Body components were printed directly onto the build plate. Bridge components were printed onto filter paper followed by cutting away excess paper to leave ~ 3.0 × 8.0 mm rectangular segments of filter paper attached to each bridge. Valves were assembled by inserting bridges into bodies. Valves were then adhered onto devices using either local heating of body components with a heat gun, adhesives (Everbuild, UK; Gorilla Glue, U.S.; Henkel, Germany), or double-sided tape (Tesa, Germany) such that the bridge components spanned a barrier.

Valves were then be reversibly operated by depressing the valve body to lock in a “flow on” state and by raising the latch to lock in a “flow off” state. Valves were tested on “dogbone” devices modified with a barrier mid-way through the channel segment over which the valve was positioned. ~ 800 µL of dye solution was pipetted onto the end of devices and allowed to flow until the solution reached the barrier.

### Reservoirs

Reservoirs consisted of two components printed separately: “reservoir” and “reservoir coupler”. Reservoir components consisted of hollow 10.0 mm diameter × 15.0 mm high cylinders for retaining solution with 1.0 mm diameter circular openings in the bottom for dispensing. Components were printed onto the build plate using PLA filament as described above for valves using slicing parameters: 0.2 mm layer height, 0.76 wall thicknesses, 0.8 bottom/top thickness, 40% infill and no build plate adhesion and a 60-degree overhang support. Reservoirs were assembled by adhering reservoir couplers to circular PP sections of “dogbone” devices as described above. To start and stop dispensation, reservoir components were then inserted into or removed from adhered reservoir couplers by hand.

### Integrated multicomponent devices

Devices consisting of one reservoir, two valves, two diamond-shaped “reaction” zones (R1 and R2), one large “sink” and a hollow octagonal junction that connected hybrid channels were fabricated as described above (Fig. 4). A washing protocol was then used to test the ability of all components to operate simultaneously. Two reservoir components containing 1.0 mL of distilled water or dye solution (~ 5% v/v blue food colouring in water) were prepared. Dye-containing reservoirs were first inserted with the valves controlling flow to R1 and R2 in the on and off states, respectively. Dye was permitted to flow until R1 was saturated with colour. The valve controlling flow to R1 was then raised into the off state, and a reservoir component containing water was inserted and allowed to wash out solution in the channel (this step was skipped for unwashed control runs). Then, the valve controlling flow to R2 was depressed into the on state and allowed to flow. Devices were then allowed to dry and reaction zones photographed while inside a white light imaging box. Dye intensities in R1 and R2 were compared following Grayscale conversion in ImageJ. Two-tailed student’s t-tests were used to compare groups with at a 0.05 level of significance. Runs were conducted in triplicate.

## Results and discussion

### Hybrid 3D printed-paper channels are easily fabricated

We developed a simple process for generating hybrid 3D printed-paper microfluidic devices (Fig. 1a). Propylene (PP) filaments were 3D printed directly onto filter paper. These substrates were then baked in an oven or hotplate. Device undersides were coated with pastel to prevent seeping during operation, but the use of single-side laminated paper could remove the need for this step altogether. This process generated hybrid channels that controlled the flow of aqueous solutions (Fig. 1b). To optimize fabrication parameters, we then printed a variety of devices with 1, 2 or 5 mm wide features, baked for 15, 30 or 45 min and baked at 150, 160, or 170 °C. The ability of channels to direct aqueous solutions without leaking through walls was then evaluated over 60 min (Fig. 1c,d). In general, wider features, longer bake times and higher bake temperatures improved device barrier integrity. At the lowest baking temperature of 150 °C, all conditions produced devices with 72% or greater failure rates. In contrast, at the highest tested bake temperature of 170 °C, devices achieved 100% barrier function when baked for 30 min or longer with 2 mm barrier widths (Fig. 1c) and when baked for 45 min at the narrowest 1 mm widths (Fig. 1d). Devices were also successfully fabricated using other polymers commonly used in 3D printing such as polylactic acid (PLA) and polycarbonate (data not shown). PP was chosen here because its relatively lower melting temperature permitted baking without discolouration of paper substrates.

Devices were developed from conceptualization to use in as little as 1 h without the need for photomasks or any specialized equipment except for a commercially available FDM printer. These print times are somewhat longer and more expensive than other commonly-used hydrophobic µPAD materials such as wax^28–32^ and ink resins^33,34^, but the numerous substrates capable of being 3D-printed allows barrier materials to be chosen based on application. For instance, materials can be printed that exhibit low bioanalyte absorption unlike polydimethylsiloxane^35^ or those that are compatible with organic solvents unlike many waxes or chemical modification methods^36^. Hydrophobic barriers are also easily fabricated without the need for expensive instruments such as vapor deposition equipment^37,38^. While we do anticipate print speeds, fabrication times and costs to decrease as 3D printing technology improves, the greatest advantage of hybrid devices in comparison to µPADs is the ability to incorporate other 3D printed elements that add valuable device functionality.

### 3D Printed elements provide intuitive fluidic control

**Figure 2:**
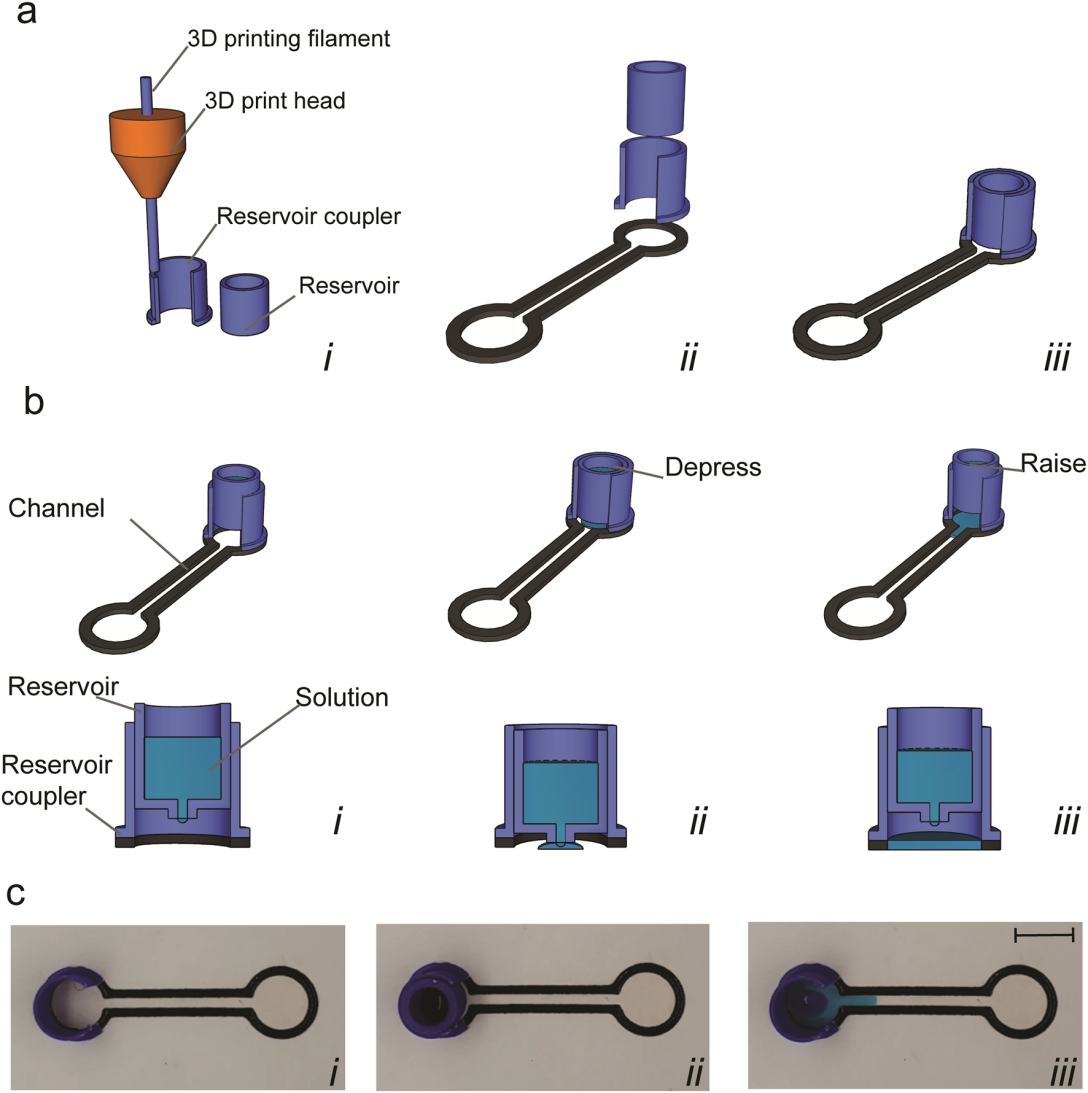
Reservoirs. (**a**) Fabrication of reservoir couplers and components by 3D printing (i), mounting couplers onto hybrid devices (ii) and slotting reservoir components into couplers (iii). (**b**) Schematic of starting dispensation by depressing reservoir component and stopping by raising. (**c**) Images of the reservoir operation showing dye dispensation. Scale bar: 10 mm.

**Figure 3:**
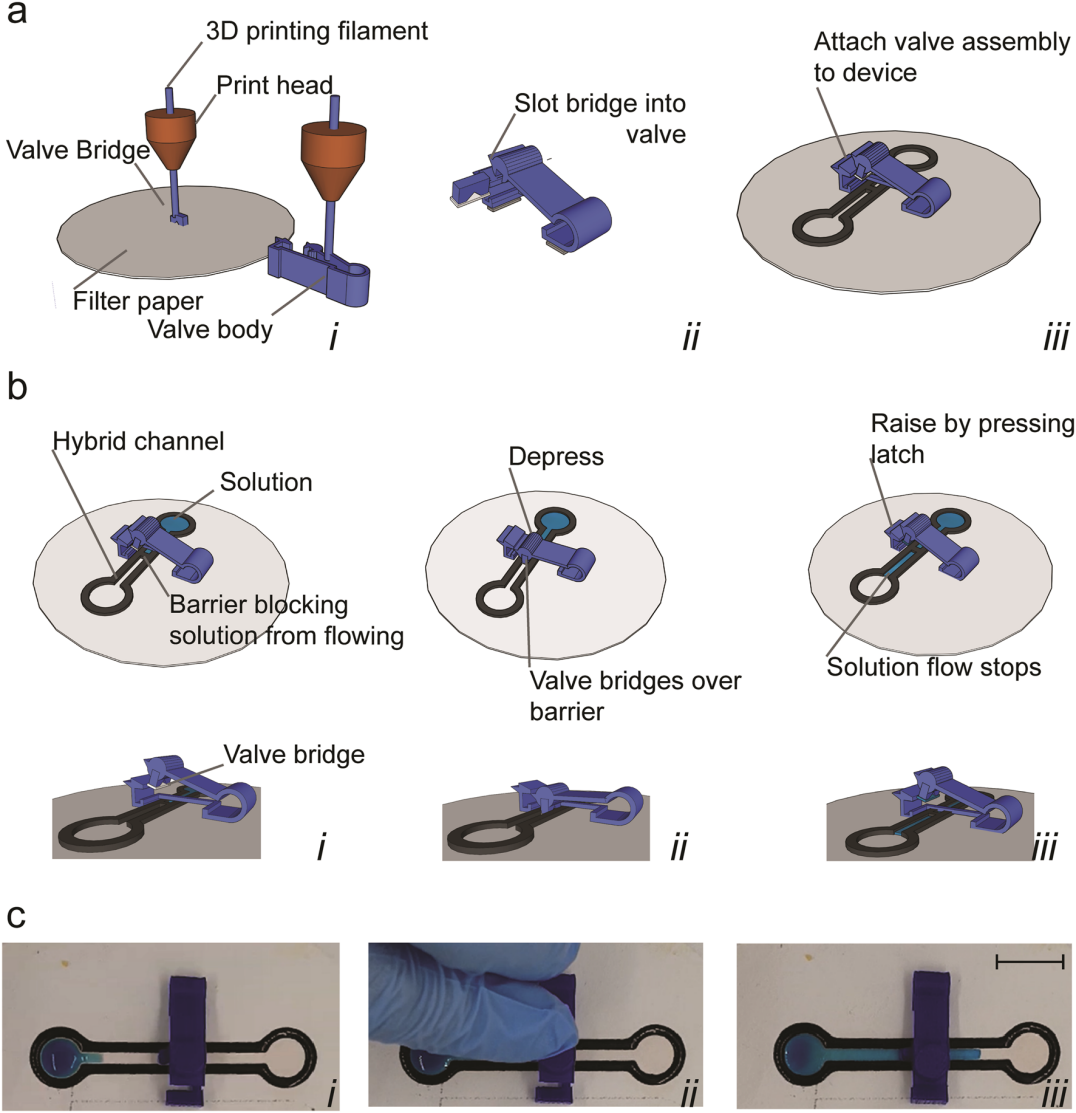
Valves. (**a**) Fabrication of valve by 3D printing bridge segment onto paper (i). Paper-laden bridge is then slotted into valve body (ii) and assembled onto a device (iii). (**b**) Schematic of finger-actuated valve operated by depressing to enable flow and raising to stop flow. (**c**) Images of valve operation showing dye solution stopped by barrier until the valve engaged by finger actuation. Scale bar: 10 mm.

The untrained operation of microfluidic devices without the need for specialized equipment is a key asset, especially in point-of-care, at-home or low-resource settings. We therefore set out to develop finger-actuated hybrid reservoirs (Fig. 2 and Supplementary Movie S1) and valves (Fig. 3 and Supplementary Movie S2) that could be intuitively operated. 3D printed cylindrical reservoirs with greater than 1 mL capacity were designed to snugly fit into couplers adhered onto circular regions of hybrid channels (Fig. 2a). Reservoirs featured 1 mm diameter holes on the bottom surfaces, narrow enough to retain aqueous solutions by surface tension, yet wide enough to rapidly dispense their contents upon depressing the reservoir (Fig. 2b,c). The ability to freely couple and decouple reservoirs from holders enabled users to easily change liquids for dispensation throughout the course of an experiment. To reduce evaporation during longer experiments or when using volatiles, we also developed lids that securely attached to the tops of reservoirs (see Supplementary information).

We then developed finger-actuated valves that consisted of valve bodies into which a hybrid 3D printed-paper bridge were slotted (Fig. 3a). Valves bodies were adhered to devices such that the paper side of bridge elements spanned hydrophobic barriers and permitted flow once depressed (Fig. 3b,c). A latching mechanism allowed valves to remain engaged once depressed and remain disengaged once raised (Fig. 3b,c). Unlike previously developed valves which have limited or no ability to reversibly switch from engaged to disengaged modes^4,6–10,12^, we have achieved over 50 actuation cycles on a single valve without failure (data not shown). Furthermore, in comparison to strategies that bypass the need for dynamic control elements such as folding “origami” devices^39,40^ or liquid actuated paper bridges^41^, hybrid valves offer versatile fluidic control and “on-the-fly” reconfigurable pathing.

The hybrid reservoirs and valves developed here require no specialized equipment for operation nor external power source since the fluid was dispensed and controlled by capillarity/hydrostatic head. These are however, just two of near-limitless potential formats and mechanisms for fluidic control possible on hybrid devices.

### Components can be integrated to increase functionality

**Figure 4:**
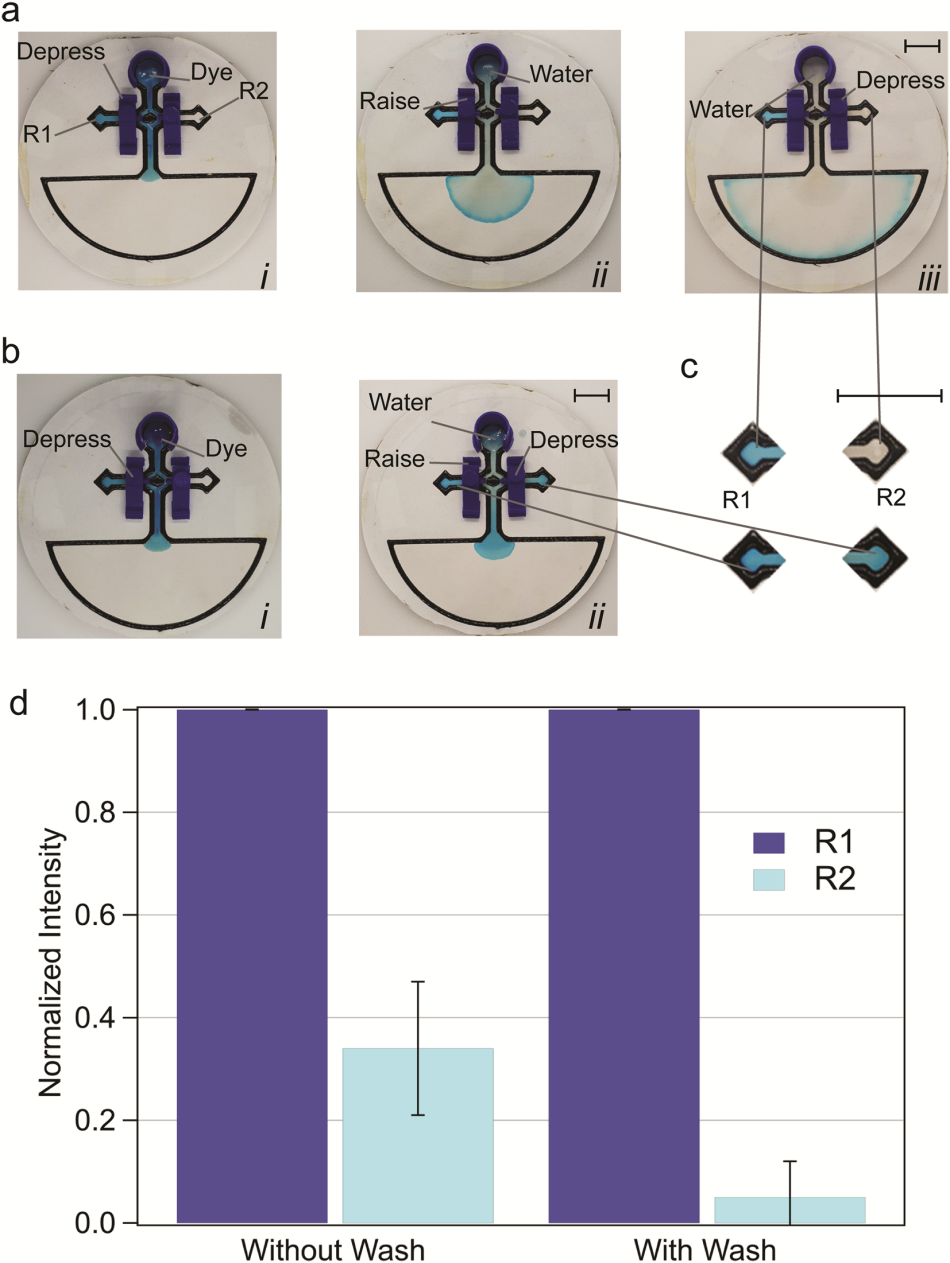
Integrated multicomponent devices. (**a**) Device wash protocol. The valve controlling flow to reservoir 1 (R1) depressed to allow flow to R1 (i) R1 valve is raised to “off” configuration and to allow wash solution flow (ii), once washed, reservoir 2 (R2) valve is depressed into the “on” configuration (**b**) Device operation without a wash step. (**c**) Dye intensities in R1 (dark blue) and R2 (light blue) with and without washing steps. Error bars represent standard deviation, N = 3. Scale bar: 10 mm.

We developed a microfluidic device that incorporated all the elements describe above to demonstrate how components can be integrated to function together. Multicomponent integrated devices consisted of one reservoir, two valves, two “reaction” zones (R1 and R2) and a large sink region for waste products (Fig. 3) all connected by hybrid channels. An important consideration was the design of the fluidic junction where channels leading to R1, R2 and sinks converged. Initial designs featured a simple cross shaped intersection that resulted in no-flow dead-end paths. These dead-end paths trapped liquids during operation leading to cross-contamination (Supplementary Fig. S1 top). We therefore updated the design with hollow octagonal intersections (Supplementary Fig. S1 bottom) to remove dead-end paths. We then conducted a wash assay using these devices to evaluate the effectiveness of control elements and to determine if hybrid channels could potentially conduct different sequential liquids with minimal cross-contamination (Fig. 4a and Supplementary Fig. S2). Dye and water solutions were successfully directed by finger operation on all devices tested. The wash protocol led to a statistically significant reduction in hybrid channel cross-contamination compared with unwashed controls (Fig. 4a–c). There was a mean 20-fold reduction in contaminating dye intensity after a single wash step versus a mean ~ threefold reduction without washing (5.0% ± 7% vs 34.0% ± 13% residual intensity, respectively). Further reductions in cross-contamination may be achievable through additional wash steps or the use of other solvents and surfactants. The ability to use a single hybrid channel for multiple sequential liquids indicates that hybrid devices may be applicable to a laboratory or analytical applications that are more complex than simple lateral flow assays.

The multicomponent integrated devices presented here capitalize on the highly complementary strengths of both 3D printed and paper-based microfluidic formats. The modular nature of the hybrid devices and their intuitive operation make them versatile for laboratory applications and well suited as teaching and outreach tools^42^. In this proof-of-concept work, integrated devices were fabricated in a stepwise manner, with reservoirs and valves adhered after device printing and baking. Fabrication times can be reduced in the future by taking advantage of differential melting temperatures of 3D printed materials. For instance, it may be possible to adhere control elements 3D printed from higher melting temperature filaments (e.g. PLA) and cure hybrid channels printed from lower melting temperature filaments (e.g. PP) in a single baking step. Furthermore, continuing advances in rapid prototyping technologies will expand the achievable resolutions and functionality of hybrid devices. Membranes, films and coverslips can be embedded directly into 3D printed components^43^ and 3D printed interfaces with analytical instruments^27^ may enable easy integration of hybrid devices with existing experimental workflows. 3D printed mechanically-compliant mechanisms^44^, “4D” time-resolved printing^45^ and the incorporation of structures that allow for the timed-release of reagents^46^ may provide additional interactive and temporal functionality in the future.

## Conclusion

Hybrid 3D printed paper devices have the potential to conduct complex experiments and point-of-care assays with no specialized equipment and minimal training. The precise fluidic control, versatile functionality and user-friendly operation provided by this technology makes it well suited to low resource or at home settings. The intuitive nature of device operation may be especially useful for self-administered point-of-care testing that reduces the burden on laboratory or healthcare systems during times of need such as the COVID-19 pandemic or other global health crises. The hybrid channels, valves and reservoirs described in this work should provide enough fluidic control to accomplish most unit operations, however more specialised components can be designed in the future to broaden the range of potential applications. For instance, through the incorporation of electrodes and ports that interface seamlessly with smartphones, mass-spectrometers or other analytical chemistry modalities; microneedles and swabs that aid the extraction of biofluids; and visual indicators for easy interpretation of results. The versatility of these devices stem from the melding of the capabilities of paper-based microfluidics with the ability of 3D printers to fabricate in free space. 3D printed and paper-based microfluidics can therefore be combined into many more configurations than we have explored here with boundless potential in future applications.

## Supplementary information

Supplementary Information 1.

Supplementary Information 2.

Supplementary Video 1.

Supplementary Video 2.
