# Supplementary material for: Hybrid 3D printed-paper microfluidics
Source: Sci Rep. 2020 Oct 27;10:18379. doi: 10.1038/s41598-020-75489-5 (PMC7591913; doi:10.1038/s41598-020-75489-5)
Supplement: Supplementary file 2 — Supplementary Information 2. [file 41598_2020_75489_MOESM2_ESM.docx]

Supplementary Information for Hybrid 3D Printed-Paper Microfluidics

Arthur Zargaryan^1^, Nathalie Farhoudi^1^, George Haworth^1^, Julian F. Ashby^1^ and Sam H. Au^1^*

^1^ Department of Bioengineering, Imperial College London, London, UK, SW7 2AZ

*Correspondence to:  s.au@imperial.ac.uk

Supplementary Figures
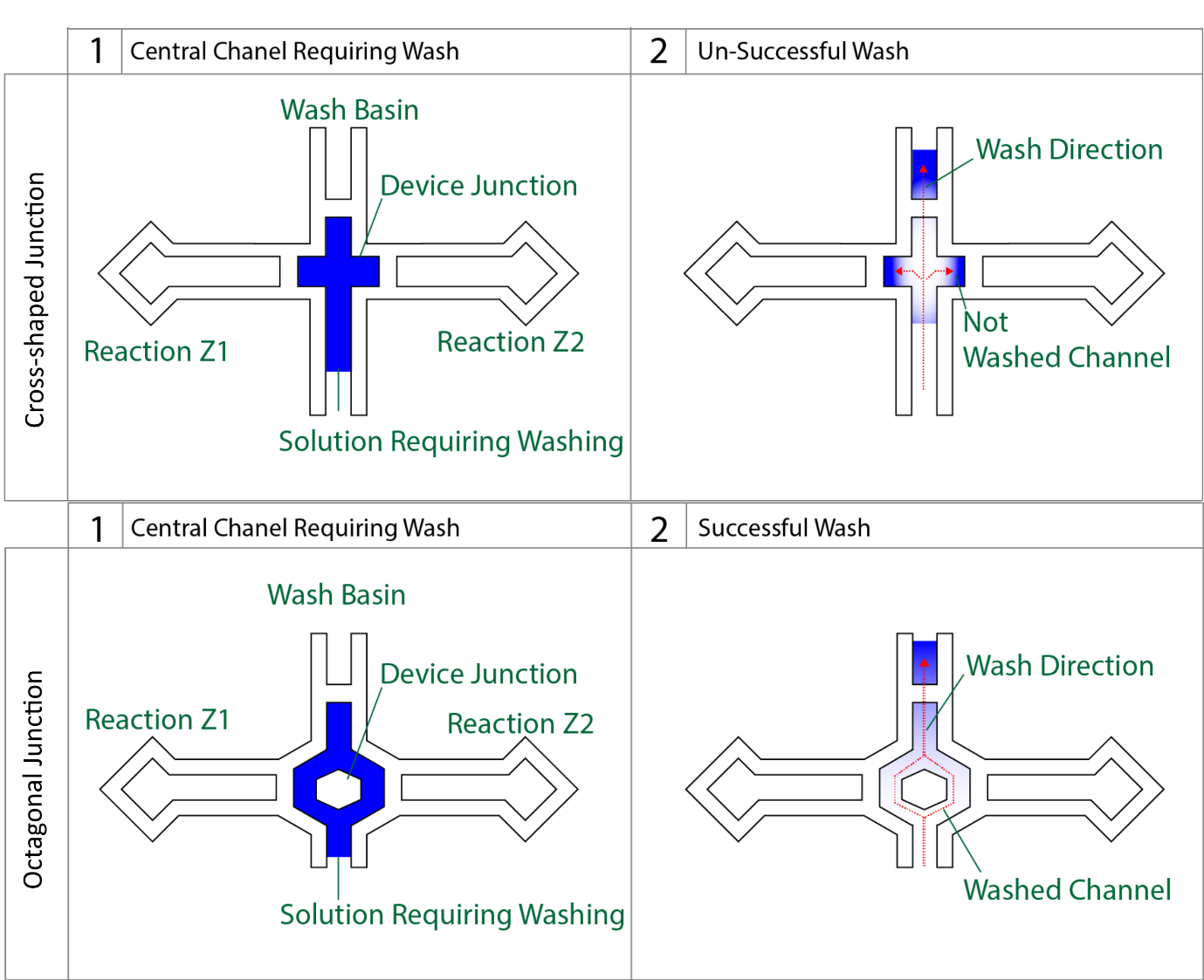


**Figure S1:** Fluidic junction design. Cross-shaped junctions led to no-flow dead-end zones that trapped fluids during wash steps (top). Octagonal junctions with hollow centres removed no-flow zones and improved wash effectiveness (bottom).


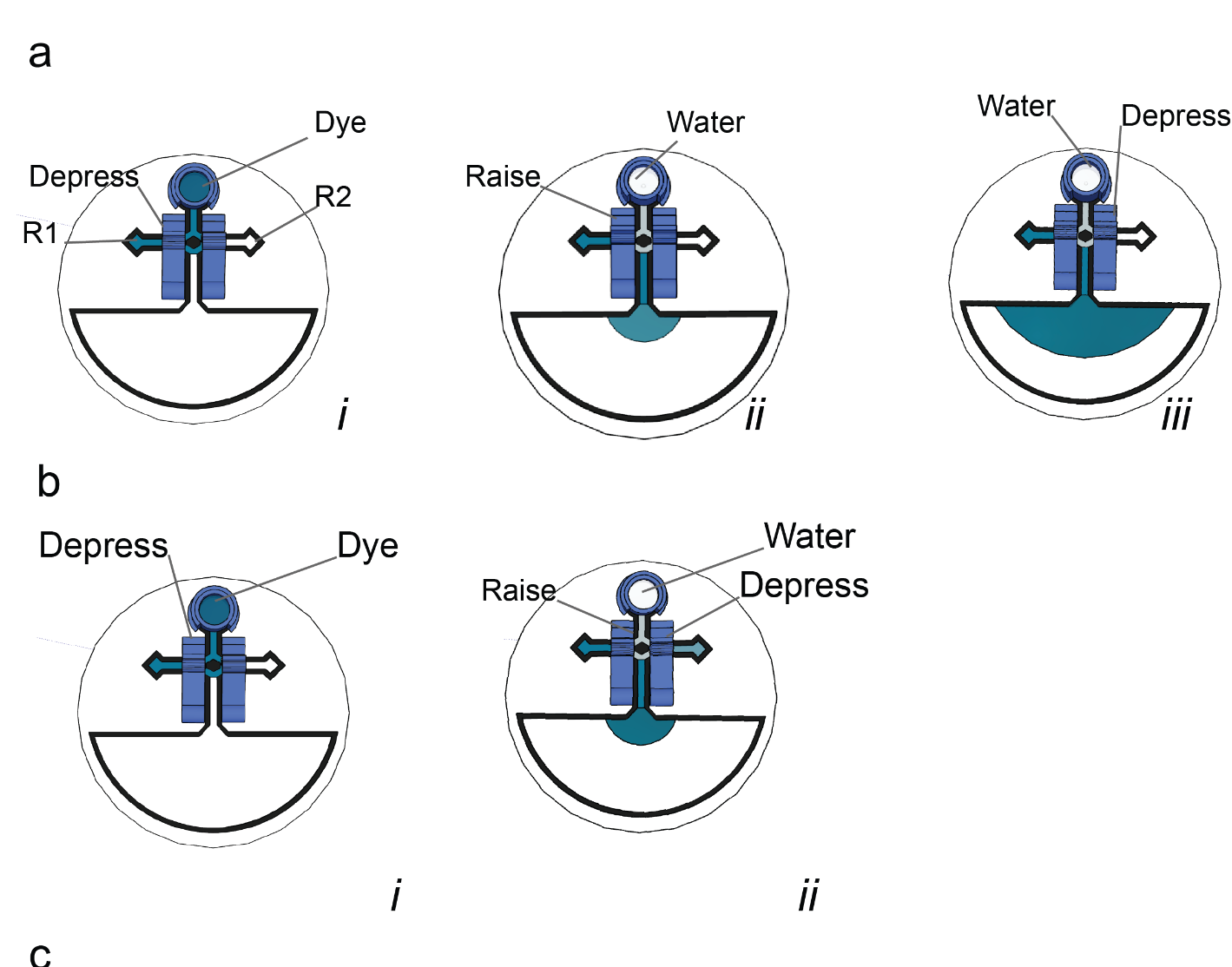


**Figure S2:** Integrated device wash protocol. (a) Wash experiment. Valve leading to reaction zone 1 (R1) depressed allowing flow of dye to R1 (i). All valves raised and water used to wash out middle channel (ii). Valve leading to reaction zone 2 (R2) depressed allowing water to flow to R2 (iii). (b) No-wash control where step ii is omitted from above.

Supplementary Movies


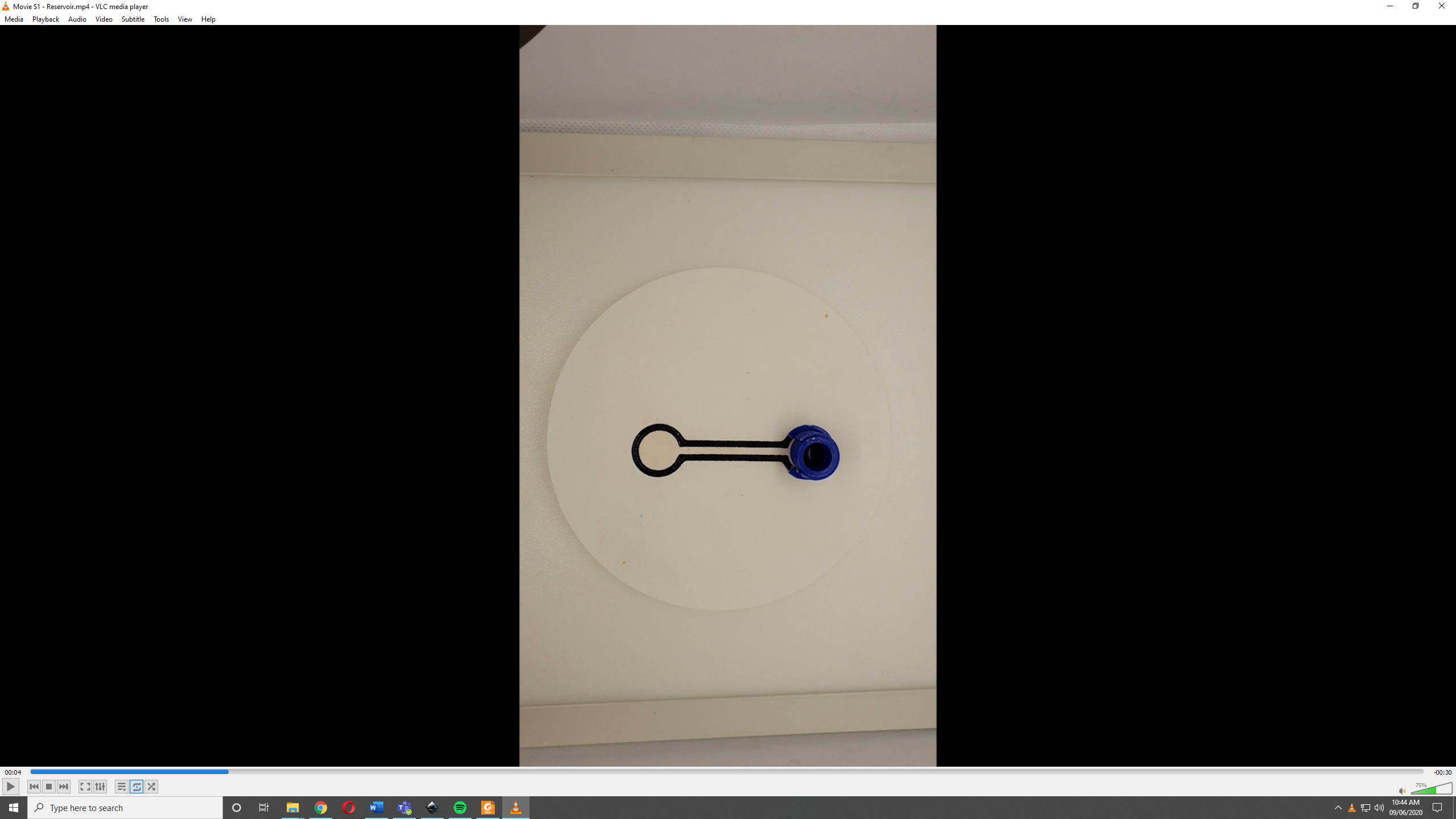


**Movie S1:** Finger-actuated operation of reservoir in hybrid 3d printed-paper device.


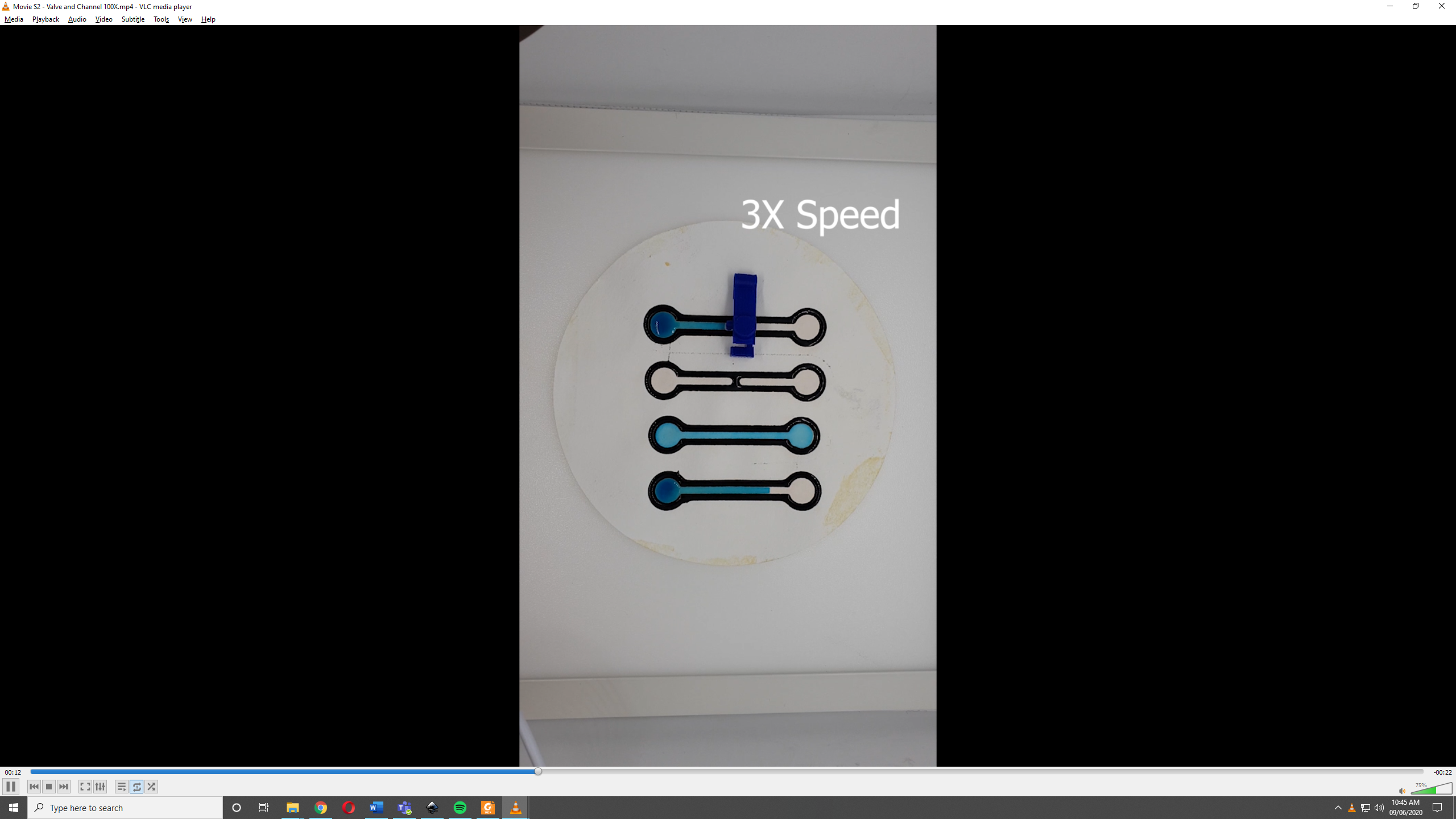


**Movie S2:** Finger-actuated operation of valves in hybrid 3d printed-paper device. Device with valve placed over barrier showing depressing to actuate “on” (top). Device with barrier but no valve or dye (2^nd^ from top). Device without barrier completely filled with dye (3^rd^ from top). Device with no barrier or valve showing unrestricted flow (bottom).

Supplementary Designs

CAD files provided as printable .STL and .3mf formats or as editable parametric Solidworks® .SLDPRT formats.

Channels:

- **Channel.STL/Channel.SLDPRT** – optimized hybrid channels
- **Testing Barrier Configurations.3mf -** Cura file with tested configurations for the channel barrier

Valve:

- **Bridge. STL/Bridge.SLDPRT -**  valve bridge component
- **Valve body.STL/Valve body.SLDPRT** - valve body component
- **Valve test.STL/Valve test.SLDPRT –**  hybrid channel configuration used to test the valve

Reservoir:

- **Reservoir.STL/Reservoir.SLDPRT –** reservoir component with a 1mm nozzle
- **Coupler.STL/Coupler.SLDPRT -** coupler that holds the reservoir in place while mounted on devices
- **Lid.STL/Lid.SLDPRT -** optional reservoir lid for reducing evaporation and contamination
- **Holder.STL/Holder.SLDPRT** - reservoir holder when reservoir not in use to prevent leakage

Integrated Device

- **Integrated.STL/Integrated.SLDPRT** – Integrated device
